# The Care of the Motor-Car Body

**Published:** 1910-06-04

**Authors:** 


### Motoring Notes.

### THE CARE OF THE MOTOR-CAR BODY.

Things learnt when young, it is said, are not forgotten in years to come. So it is with observations and impressions. Everyone will remember that the old family coachman would never allow dirt or mud to dry on the carriage after returning from a wet drive. This is an object-lesson, and in it lies the crux of the whole situation comprised in the subject heading this article.

The touring season will soon be upon us, with its varying weather. Prospective owners will soon be taking deliveries of new cars, and old owners be receiving theirs back with new coats of paint and varnish fresh upon them, when it is, more than at any other time, that caution in cleaning and washing is necessary. The outward appearance of many a car has been ruined the first time of washing, partly from inexperience, but not necessarily so, but oftentimes because the new surfaces had not really hardened, the owners being anxious to try the newly acquired purchase or to renew acquaintance with an old friend.

It is for the benefit of the former that these remarks are specially penned, and if properly digested there is no reason why the fortunate possessor of a motor vehicle should not be able to tell at a glance if the old hand or newly engaged chauffeur is going the right way to work or not, and, if the latter, to correct him in the error of his ways. At the body-builder's the paint-shops are kept at an even, warm temperature, to suit the varying operations in hand and the convenience of the men employed. Such an atmosphere is not always the best one for hardening off. If the car is one only recently built or repainted it should be taken home on a dry day, paper having been first placed round the panels to keep off dust or dirt. The car should then be washed carefully as afterwards described, then left alone for a week or so, after which it should be washed daily for fourteen days with cold water.

It is not proposed to deal with the engine work, so that need not be brought into the compass. In regard to the body the main features to be avoided are—never to allow, if possible, dirt to dry on, and, if that has taken place, under no circumstances make any attempt to remove it in its chrysalis state. Dust is equally as effective in scratching the polished face if removed dry; this can be readily understood when it is realised that fine dust is powdered flint or equally hard substances, with cutting edges like broken glass, which it resembles under a magnifying glass. The old saying "Spare the rod and spoil the child" may well be converted to "Spare the water, spoil the appearance of the car." Having removed the lamps, mats, covered up the upholstery and brushed out the footboards, etc., sponge the hood and leatherwork with a clean sponge. Then if a hose is available, attach a rose with fine holes, and apply the water by holding the rose near the top of the panelled part and allow the water to flow downwards. Under no consideration should it be dashed on in a direct stream under pressure, as if a fire were being extinguished. Such action is as fatal as an attempt to remove the objects desired when half wet with a sponge.

If no hose is available, use a garden syringe or watering-can with a rose. When the top part of the car is quite freed from all dirt, the same may be done with the wheels, but more water pressure may be used. Apply a soft sponge free from grit to the whole, and finish off with a soft cloth or silk rag. A spoke brush may be usefully employed on the wheels, but not, as one oftentimes sees, banged up and down from hub to felloe. This not only injures the paint, but also the wheels; such an action is so totally different from that which a wheel meets with in ordinary wear that in time it will, and does, loosen the spoke ends in the rims. A duplicate set of washing and cleaning materials should be used, one set for the body and the other for the wheels and underpart. This is absolutely necessary, because it is impossible to free those used for the wheels from grease, and either that or oil, particularly lubricating oil (to distinguish such from petrol, etc.), is very detrimental to all painted and varnished surfaces.

The tyres should be well dried and freed from all oil, grease, etc., as anything of this nature is highly injurious to rubber. The brass or plated work can best be cleaned by some of the well-known preparations sold for the purpose, paste being preferable to powder. The leatherwork should be rubbed over with an oily rag; if after much use more is necessary. This can then be polished with a clean soft duster. If one does not care to use the oily rag there are many preparations on the market for cleansing, polishing, and preserving leather, any of which can safely be employed. If the car is fitted with a collapsible hood, whether of the cape or leather Victoria types, always leave it up when in the garage, otherwise the creases will become permanent marks and in time cause cracks. The lamps, needless to say, should be refilled and trimmed after each journey if they have been used, and always stored in a cupboard away from damp and dust. A frequent source of trouble arises even if the car is well sheeted up, and the entrance doors kept closed, from the dust that blows in underneath. A good plan is to have a loose board made to fit inside the door uprights and slip in between some slots, so that it can readily be removed and replaced.

